# Image-Guided Versus Blind Corticosteroid Injections in Adults With Shoulder Pain: A Systematic Review and Meta-Analysis

**DOI:** 10.7759/cureus.17032

**Published:** 2021-08-09

**Authors:** Peri Harish Kumar, Tajbinder Singh Bains, Naveen Shejale, Varinder Kaur

**Affiliations:** 1 Department of Surgery, Armed Forces Medical College, Pune, IND; 2 Department of Orthopedics, Indian Naval Hospital Ship Asvini, Mumbai, IND; 3 Department of Surgery, Adesh Institute of Medical Sciences and Research, Bathinda, IND

**Keywords:** corticosteroid, shoulder pain, blind injections, image-guided injection, meta-analysis

## Abstract

The present study aimed to compare the clinical effects of image-guided versus blind steroid injection to treat shoulder pain, which is always debatable. An electronic search of credible databases was conducted for randomized controlled trials (RCTs) comparing image-guided versus blind steroid injections. The continuous data were pooled as mean difference (MD) or standardized mean difference (SMD), and dichotomous data were grouped as odds ratio (OR) with 95% confidence interval (CI). Sensitivity analysis and leave-one-out analysis were performed. The meta-analysis of 20 RCTs comprising 1136 patients favored image-guided injection based on the MD of the visual analog score (VAS) and shoulder function scores, measured between six weeks follow-up and baseline (MD=0.63, 95% CI [0.13, 1.12], p=0.01 and SMD=0.35, 95% CI [0.05, 0.65], p=0.02, respectively). Meta-analysis did not favor either group regarding the shoulder disability scores or side effects (MD=-2.18, 95% CI [-12.19, 7.83], p=0.67, and OR=0.40, 95% CI [0.14, 1.15], p=0.09, respectively). The image-guided approach was associated with significant improvement in pain and shoulder functionality. However, no significant difference was observed between the two approaches in terms of disability scores and side effects.

## Introduction and background

Shoulder pain can cause significant morbidity and compromise the person's ability to carry out daily activities and work [1-2]. The prevalence of shoulder disorders ranges from 7% to 36%, and shoulder pain is prevalent in 16% to 26% of the population, and hence considered the third most common cause of musculoskeletal problems in primary care. Up to 1% of adults consult a general practitioner with the new incidence of shoulder pain annually [1,3]. Causes of shoulder pain include subacromial impingement syndrome, rotator cuff syndrome, subacromial bursitis, adhesive capsulitis, and biceps tendinitis [4].

Irrespective of the underlying etiology, glucocorticoid injections are widely used to treat shoulder pain and improve the functionality of shoulder movements. Two methods are used for drug delivery, a landmark-guided approach (i.e. blind) and an image-guided approach using ultrasonography [5]. The landmark-guided approach is ruled by factors such as local anatomy, distribution of peripheral fat, body mass index, different lengths of needles used, a suitable technique, and experience of the operator. Using ultrasound-guided steroid injection comes with the drawbacks of added cost, the need for an experienced operator, and it is time-consuming [6-7].

A comprehensive study is required to assess the evidence available and determine the best approach for managing shoulder pain. In our systematic review and meta-analysis, we investigated the efficacy and safety of image-guided versus blind corticosteroid injections in adults with shoulder pain.

## Review

Material and methods

According to the Cochrane Handbook of Systematic Reviews of Interventions and the Preferred Reporting Items for Systematic Reviews and Meta-Analyses (PRISMA) statement guidelines, we carried out this study. No institutional review board approval was required for this systematic review.

Search Strategy

Relevant studies were identified through an extensive electronic search of databases such as PubMed, Web of Science, and Cochrane CENTRAL for reports till June 2021 from the inception. The search terms were (blind OR landmark OR anatomical OR image-guided OR ultrasound OR fluoroscopy) AND (steroid OR corticosteroid OR glucocorticoid OR triamcinolone OR methylprednisolone OR hydrocortisone OR prednisolone OR cortisone OR dexamethasone OR betamethasone) AND (frozen shoulder OR adhesive capsulitis OR shoulder pain OR shoulder impingement syndrome OR rotator cuff OR bursitis). Additionally, the reference lists of included studies were screened manually to identify additional articles, that are potentially relevant.

Study Selection

We included randomized controlled trials (RCTs) according to the PICO approach: P, patients with shoulder pain; I, ultrasound-guided corticosteroid (US-guided CS); C, comparison with blind or landmark injection of CS; and O, efficacy outcomes including changes of the visual analog score (VAS), shoulder function scores, shoulder abduction between baseline and six weeks follow-up, and safety outcomes included side effects. Exclusion criteria were observational studies, review articles, case reports, comments or guidelines, animal studies, non-English articles, and insufficient data to calculate. Disagreements were solved by consensus.

Data Extraction

For each paper, we extracted the following information: name of the first author, year of publication, country, number of patients, their mean age, percentage of females, type of shoulder disease, follow-up durations, and efficacy and safety outcomes.

Quality Assessment and Data Extraction

We used Cochrane Collaboration's tool for assessing the risk of bias of included RCTs [8]. Risk of bias assessment included the following domains: 1) sequence generation, 2) allocation sequence concealment, 3) blinding of participants and personnel, 4) blinding of outcome assessment, 5) incomplete outcome data, 6) selective outcome reporting, and 7) other potential sources of bias; the authors' judgment is categorized as 'Low risk,' 'High risk,' or 'Unclear risk' of bias.

Statistical Analysis

Continuous data were calculated as mean difference (MD) or standardized mean difference (SMD) with a 95% confidence interval (CI). Dichotomous data were pooled as odds ratio (OR), with 95% CI. Heterogeneity was assessed by visual inspection of the forest plots and measured by Q statistics and statistics. Significant statistical heterogeneity was indicated by a Q statistic P-value less than 0.1 or by more than 50%. In case of significant heterogeneity, a random effect model was employed. Otherwise, the fixed-effect model was used. All statistical tests were two-sided, and P < 0.05 was considered statistically significant. Leave-one-out-meta-analysis was performed to assess the contribution of each study to the overall model. The meta-analysis of all studies enrolled was conducted in OpenMeta[analyst] software.

Results

Search Strategy

**Figure 1:**
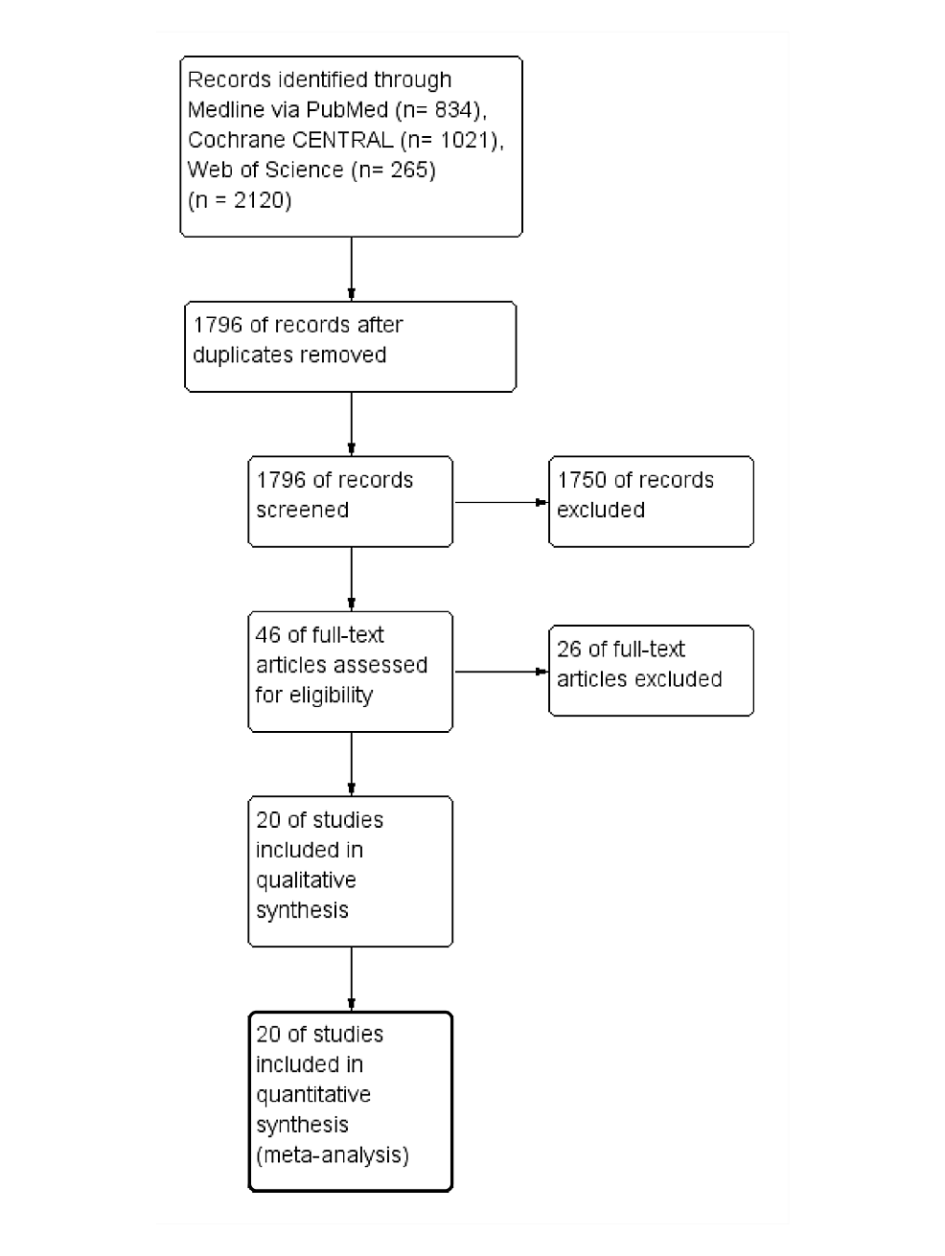
The PRISMA flow diagram of study selection PRISMA: Preferred Reporting Items for Systematic Reviews and Meta-Analyses

A total of 2120 relevant papers were initially identified using the search strategy. A total of 324 duplicated papers were excluded by EndNote software, along with another 1750 papers that did not fit the criteria for analysis on the title and abstract screening. Based on full-text screening, 20 articles were finally included. The PRISMA flow process of study selection is shown in Figure 1.

Characteristics and Quality Assessment of the Included Studies

Ultimately, 20 RCTs, conducted in a total of 1136 patients, were included in this meta-analysis [9-28]. Of them, 581 patients had US-guided CS, and 555 had landmark CS injection. The patients included were diagnosed with subacromial impingement syndrome, rotator cuff syndrome, subacromial bursitis, adhesive capsulitis, and biceps tendonitis. All studies were published in English during the years from 2004 to 2021. Table 1 describes the significant characteristics of enrolled patients in each RCT.

**Table 1:** Summary table of studies included showing the significant characteristics of enrolled patients in each RCT T: treatment; C: control; US-guided, ultrasound-guided; CS, corticosteroid; RCT, randomized controlled trial; DASH, the Disabilities of the Arm, Shoulder, and Hand; ASES, the American Shoulder and Elbow Surgeons score; CMS, Constant-Murley Score; ADLs, activities of daily living; SDQ, Shoulder Disability Questionnaire; SPADI, Shoulder Pain and Disability Index; SF-36, the 36-item Short-Form Health Survey

Author	Year	Country, and study design	Number of patients	Age, mean	% of Females	Diagnosis	Follow-up (weeks)
Cho et al. [24]	2021	South Korea, RCT	T: 45; C: 45	-	-	Primary frozen shoulder	12
Akbari et al. [21]	2020	Turkey, RCT	T: 14; C: 14	T: 40.75; C: 42.25	T: 57.1; C: 64.3	Subacromial impingement syndrome	4
Yiannakopoulos et al. [19]	2020	Greece, RCT	T: 22; C:22	T: 41.5; C: 43.9	T: 54.5; C: 40.9	Bicipital tendinosis	6
Raeissadat et al. [28]	2020	Iran, RCT	T: 20; C: 21	T: 57.8; C: 59.9	T: 35; C: 38.1	Shoulder adhesive capsulitis	4
Bhayana et al. [20]	2018	India, RCT	T: 30; C: 30	T: 44.53; C: 42.03	T: 56.6; C: 33.3	Rotator cuff syndrome	12
Coene et al. [9]	2017	USA, RCT	T: 9; C: 11	54	T: 50; C: 50	Shoulder adhesive capsulitis	12
Cole et al. [17]	2015	Australia, RCT	T: 28; C: 28	T: 46; C:42	T: 50; C: 64	Subacromial impingement syndrome	6
Haghighat et al. [11]	2015	Iran, RCT	T: 20; C:20	T: 50.45; C: 52.3	T: 60; C: 65	Subacromial impingement syndrome	6
Saeed et al. [18]	2014	Ireland, RCT	T: 50; C: 50	57.7	65	Subacromial impingement syndrome	12
Hsieh et al. [26]	2013	Taiwan, RCT	T: 46; C: 46	T: 57.59; C: 55.87	T: 58.7; C: 63	Subacromial bursitis	4
Dogu et al. [22]	2012	Turkey, RCT	T: 23; C: 23	T: 55.17; C: 56.74	T: 65.2; C: 69.6	Subacromial impingement syndrome	6
Zufferey et al. [16]	2012	Switzerland, RCT	T: 32; C: 33	T: 53; C: 54	T: 40.6; C: 45.5	Subacromial bursitis	6
Hashiuchi et al. [15]	2011	Japan, RCT	T: 15; C: 15	T: 59.7; C: 67.8	T: 46.7; C: 66.7	Biceps tendon inflammation	-
Zhang et al. [14]	2011	China, RCT	T: 53; C: 45	T: 49; C: 43	T: 35.8; C: 35.6	Biceps tendon inflammation	32.5
Panditaratne et al. [12]	2010	United Kingdom, RCT	T: 41; C: 17	54	62.1	Subacromial bursitis	8
Ekeberg et al. [10]	2009	Norway, RCT	T: 53; C: 53	T: 51; C: 50	T: 60; C: 62	Rotator cuff disease	6
Lee et al. [25]	2009	South Korea, RCT	T: 20; C: 20	T: 53.1; C: 54.1	T: 55; C: 50	Shoulder adhesive capsulitis	6
Ucuncu et al. [23]	2009	Turkey, RCT	T: 30; C: 30	T: 52.1; C: 52.9	T: 73.3; C: 73.3	Impingement syndrome	6
Chen et al. [27]	2006	Taiwan, RCT	T: 20; C: 20	53	33.3	Subacromial bursitis	1
Gomoll et al. [6]	2004	Spain, RCT	T: 21; C: 20	T: 52.9; C: 51.9	T: 71; C: 60	Impingement syndrome	6

**Figure 2:**
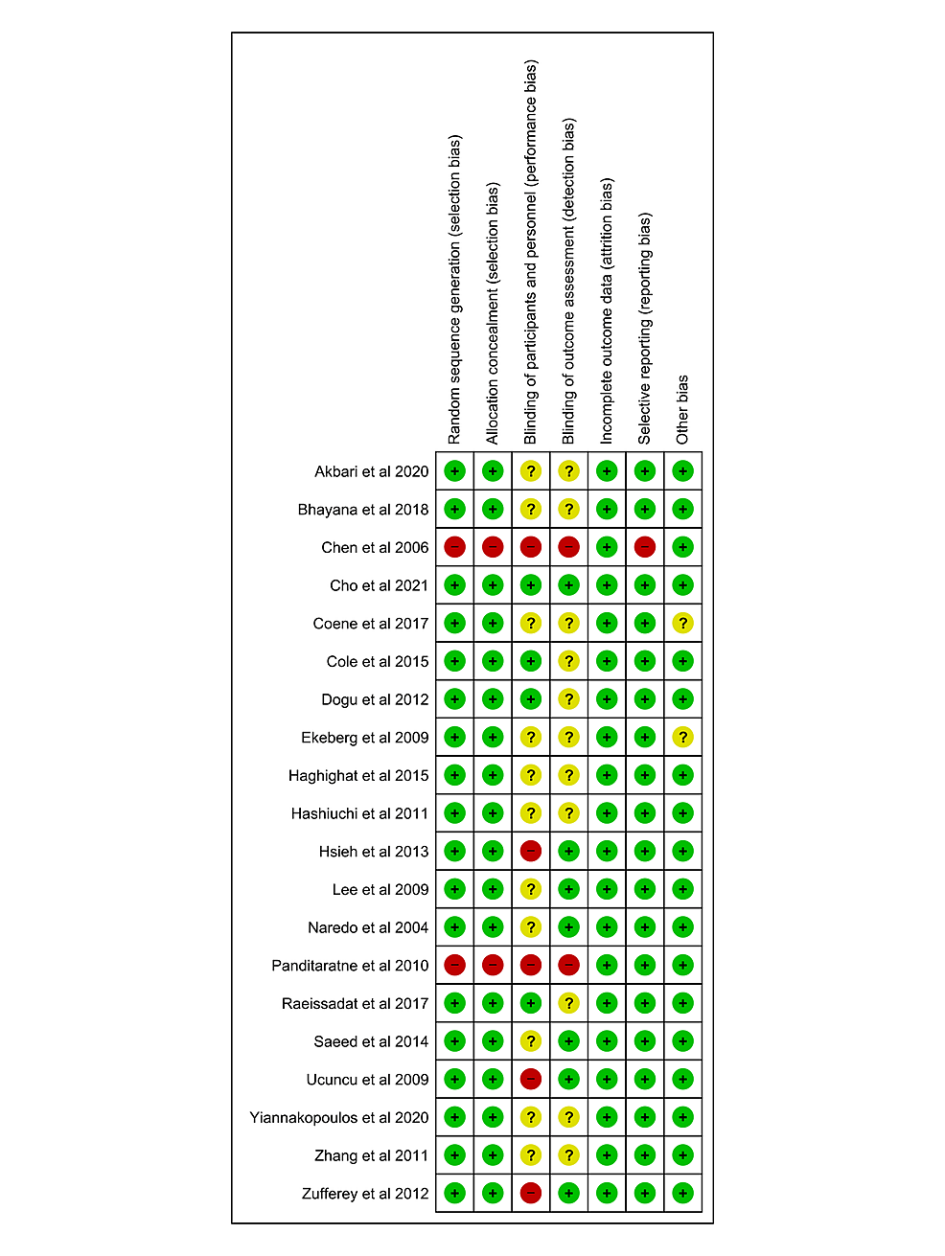
The summary of the risk of bias assessment of include studies Included studies are Akbari et al. [21], Bhayana et al. [20], Chen et al. [27], Cho et al. [24], Coene et al. [9], Cole et al. [17], Dogu et al. [22], Ekeberg et al. [10], Gomoll et al. [6], Haghighat et al. [11], Hashiuchi et al. [15], Hsieh et al. [26], Lee et al. [25], Panditaratne et al. [12], Raeissadat et al. [28], Saeed et al. [18], Ucuncu et al. [23], Yiannakopoulos et al. [19], Zhang et al. [14], Zufferey et al. [16].

The summary of the risk of bias assessment is shown in Figure 2.

Outcomes

Visual Analogue Score (VAS)

**Figure 3:**
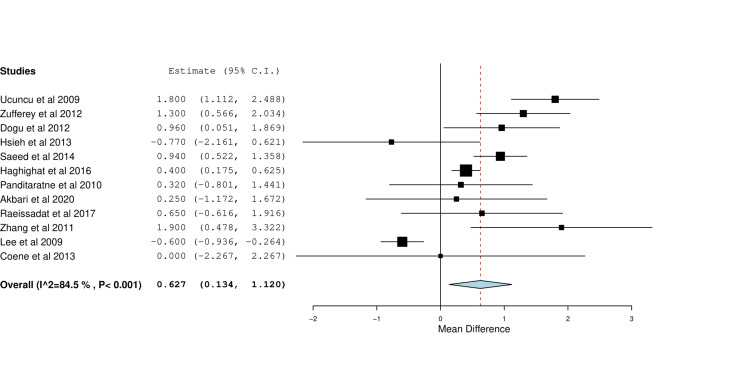
Forest plot of mean changes of visual analog score (VAS) between baseline and six weeks of image-guided vs blind steroid injection Included studies are: Dogu et al. [22], Ucuncu et al. [23], Zufferey et al. [16]. Haghighat et al. [11], Hsieh et al. [26], Saeed et al. [18], Akbari et al. [21], Panditaratne et al. [12], Raeissadat et al. [28], Coene et al. [9], Lee et al. [25], Zhang et al. [14]

Twelve studies reported data on changes in VAS scores between baseline and six weeks. Meta-analysis showed that US-guided CS injection provided significant reductions in VAS score than landmark injection (MD=0.63, 95% CI [0.13, 1.12], p=0.01). Significant heterogeneity was observed (I^2^=85%, P < 0.001) (Figure 3).

Shoulder Function Scores

**Figure 4:**
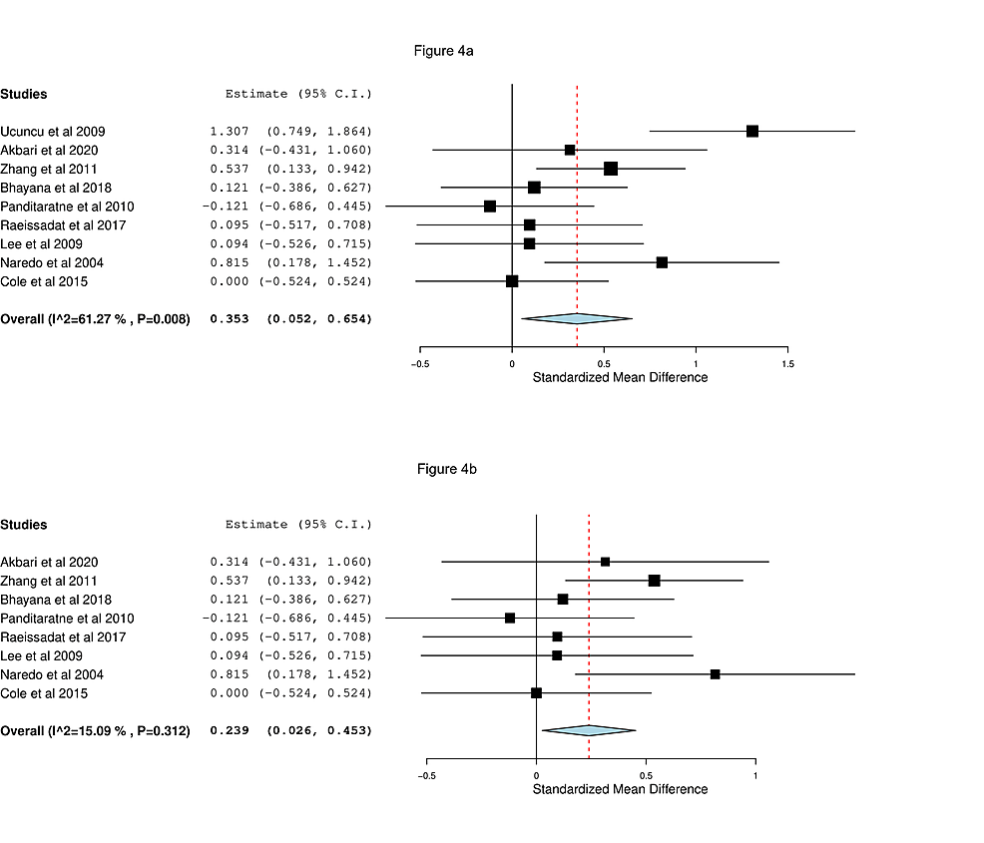
Forest plots of (a) mean changes and (b) sensitivity analysis of mean changes of shoulder function scores between baseline and six weeks of image-guided vs blind steroid injection. Included studies are: Akbari et al. [21], Bhayana et al. [20], Cole et al. [17], Gomoll et al. [6], Lee et al. [25], Panditaratne et al. [12], Raeissadat et al. [28], Ucuncu et al. [23], Zhang et al. [14].

Nine studies reported on changes in shoulder function scores between baseline and six weeks. Four of them used the Constant-Murley Score, two studies used the Oxford score, and three studies used other scores. Meta-analysis showed that US-guided CS was associated with better shoulder function scores at six weeks than landmark CS (SMD=0.35, 95% CI [0.05, 0.65], p=0.02) as shown in Figure 4a. Significant heterogeneity was observed (I^2^=61%, p=0.008), which was resolved after excluding the study by Ucuncu et al. (2009) (SMD=0.24, 95% CI [0.03, 0.45], p=0.03; I^2^=15%, p=0.31), as shown in Figure 4b.

Shoulder Pain and Disability Index (SPADI)

**Figure 5:**
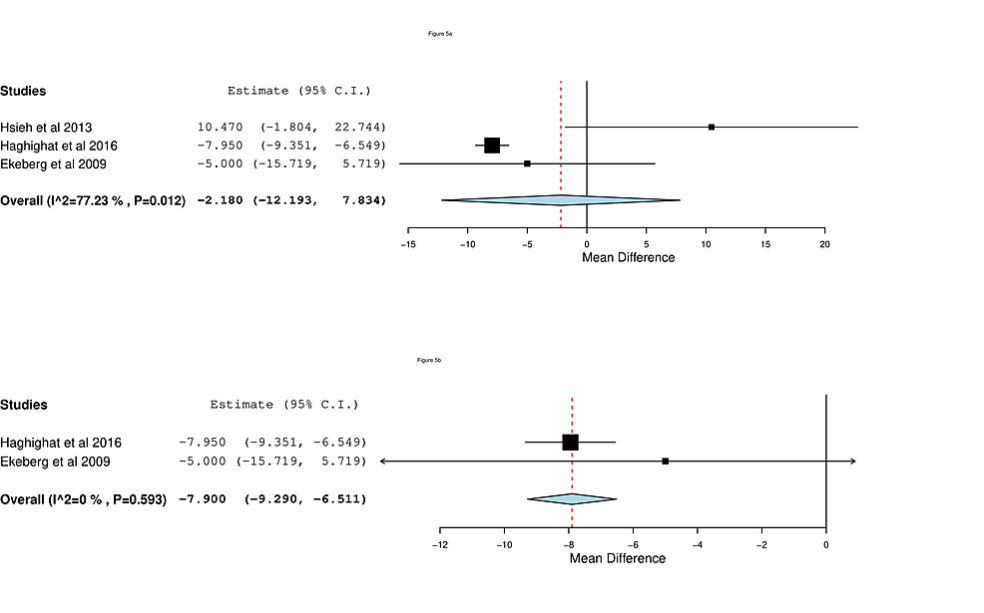
Forest plots of (a) mean changes of SPADI and (b) sensitivity analysis of mean changes of SPADI between baseline and six weeks of image-guided vs blind steroid injection. Included studies are: Ekeberg et al. [10], Haghighat et al. [11], Hsieh et al. [26].

Three studies reported on changes of SPADI at the six weeks follow-up. Meta-analysis did not favor either group (MD=-2.18, 95% CI [-12.19, 7.83], p=0.67), as represented in Figure 5a. Significant heterogeneity was observed (I^2^=77%, P < 0.012). Heterogeneity was resolved after excluding the study by Hsieh et al. (2013) (MD=-7.9, 95% CI [-9.29, -6.15], P < 0.001; I^2^=0%, p=0.59), which is shown in Figure 5b.

Shoulder Abduction Degree

**Figure 6:**
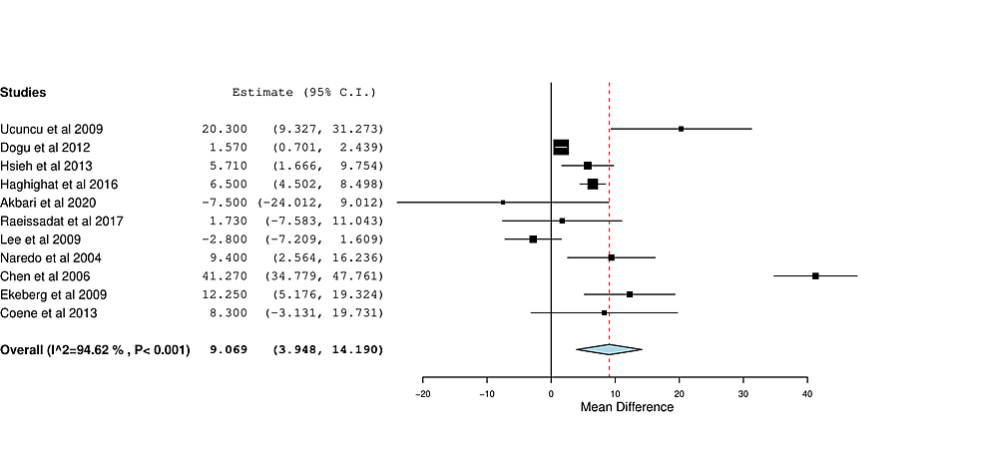
Forest plot of mean changes of shoulder abduction degree between baseline and six weeks of image-guided vs blind steroid injection. Included studies are: Akbari et al. [21], Coene et al. [9], Dogu et al. [22], Ekeberg et al. [10], Haghighat et al. [11], Hsieh et al. [26], Lee et al. [25], Raeissadat et al. [28], Ucuncu et al. [23], Gomoll et al. [6].

Eleven studies reported on changes in shoulder abduction at six weeks. Meta-analysis showed that US-guided CS was associated with larger shoulder abduction than landmark CS (MD=6.07, 95% CI [3.95, 14.19], P < 0.001). Significant heterogeneity was observed (I^2^=95%, P < 0.001) and is shown in Figure 6.

Adverse Events

**Figure 7:**
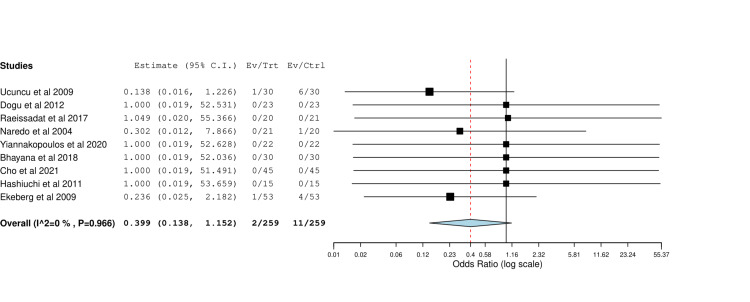
Forest plot of side effects of image-guided vs blind steroid injection Included studies are: Dogu et al. [22], Raeissadat et al. [28], Ucuncu et al. [23], Bhayana et al. [20], Cho et al. [24], Ekeberg et al. [10], Gomoll et al. [6], Hashiuchi et al. [15], Yiannakopoulos et al. [19].

Nine studies have reported on adverse events, including infection and skin peeling. Meta-analysis showed no significant difference between the compared groups (OR=0.40, 95% CI [0.14, 1.15], p=0.09), and significant heterogeneity was observed (I^2^=0%, p=0.97), and are shown in Figure 7.

Leave-One-Out-Analysis

**Figure 8:**
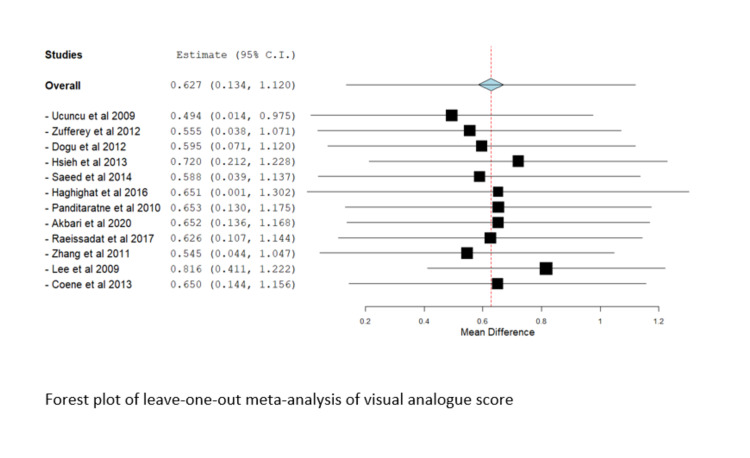
Forest plot of leave-one-out meta-analysis of the visual analog score Included studies are: Dogu et al. [22], Ucuncu et al. [23], Zufferey et al. [16]. Haghighat et al. [11], Hsieh et al. [26], Saeed et al. [18], Akbari et al. [21], Panditaratne et al. [12], Raeissadat et al. [28], Coene et al. [9], Lee et al. [25], Zhang et al. [14].

Significant heterogeneity was observed in most outcomes (I^2^ > 50%, P < 0.1); therefore, a random-effects model was utilized. We conducted a leave-one-out meta-analysis to assess the contribution of each study to the overall model. No study affected the overall estimate in the leave-one-out-study analysis, and there was no marked difference in results, suggesting that the results of the current study were not driven by a single study or a supplementary file. The Forest plot of the leave-one-out meta-analysis of the visual analog score is shown in Figure 8.

**Figure 9:**
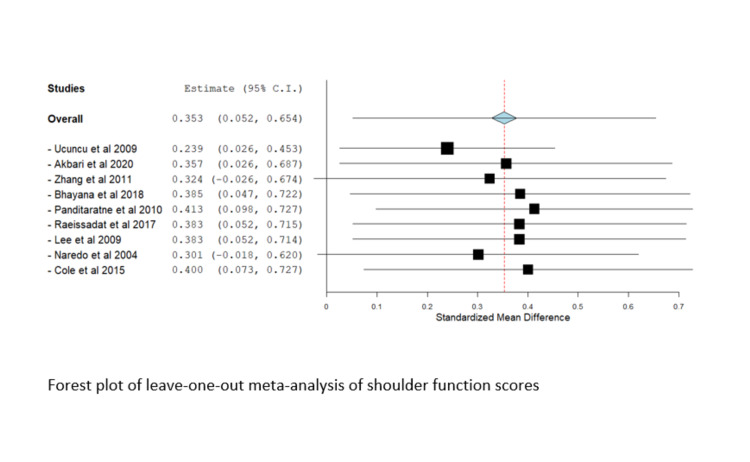
Forest plot of leave-one-out meta-analysis of shoulder function scores. Included studies are: Akbari et al. [21], Bhayana et al. [20], Cole et al. [17], Gomoll et al. [6], Lee et al. [25], Panditaratne et al. [12], Raeissadat et al. [28], Ucuncu et al. [23], Zhang et al. [14].

Figure 9 represents the Forest plot of the leave-one-out meta-analysis of shoulder function scores.

**Figure 10:**
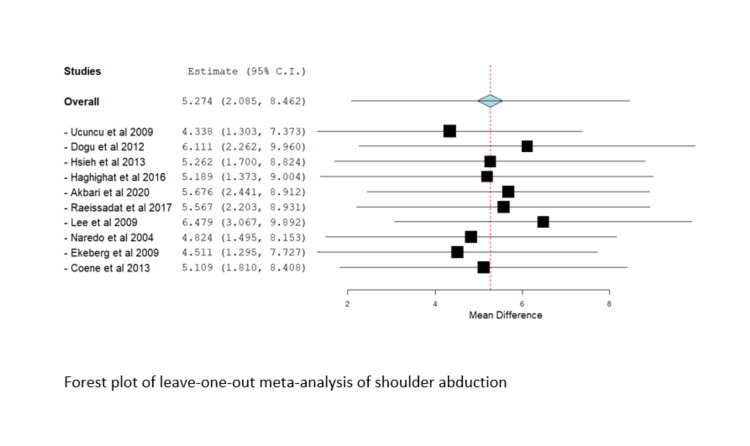
Forest plot of the leave-one-out meta-analysis of shoulder abduction Included studies are: Akbari et al. [21], Coene et al. [9], Dogu et al. [22], Ekeberg et al. [10], Haghighat et al. [11], Hsieh et al. [26], Lee et al. [25], Raeissadat et al. [28], Ucuncu et al. [23], Gomoll et al. [6].

As discussed, the Forest plot of leave-one-out meta-analysis of shoulder abduction can be found in Figure 10.

**Figure 11:**
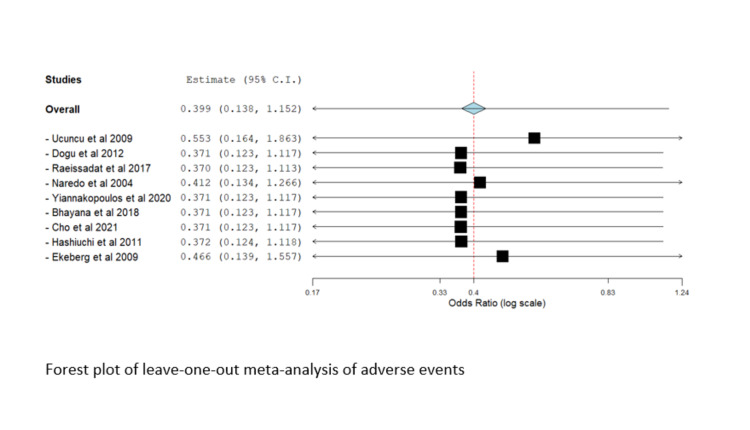
Forest plot of the leave-one-out meta-analysis of adverse events Included studies are: Dogu et al. [22], Raeissadat et al. [28], Ucuncu et al. [23], Bhayana et al. [20], Cho et al. [24], Ekeberg et al. [10], Gomoll et al. [6], Hashiuchi et al. [15], Yiannakopoulos et al. [19].

Figure 11 shows the Forest plot of the leave-one-out meta-analysis of adverse events.

Discussion

The current meta-analysis showed that image-guided steroid injection was associated with a definite improvement in pain and shoulder functionality. However, the difference between the two approaches was not significant regarding disability scores and side effects.

Our results are concordant with two previously reported meta-analyses [29-30]. A meta-analysis by Aly et al. reported their results based on three studies for the subacromial space and found that the image-guided group had a significant reduction in pain compared to the landmark-guided (LMG) group at six weeks post-injection [30]. Intriguingly, the accuracy of needle injection was similar between the two groups (70% in the blind injection group and 65% in the image-guided group). On the other hand, improved accuracy of the glenohumeral joint and biceps tendon sheath injections was associated with an improved clinical outcome in the US group [30]. Another meta-analysis by Bloom et al. included three studies that reported pain improvement in the image-guided group compared to the blind injection group. There was significant heterogeneity, and when the sensitivity analysis is carried out, the benefit was no longer apparent [29].

Many scores assess functional outcomes of the shoulder, and our analysis showed that image-guided steroid injection was resulted in better shoulder function scores at six weeks compared to blind steroid injection. Aly et al. reported an improvement in the shoulder function in the image-guided group at six weeks post-injection compared to the blind injection group. However, there was significant heterogeneity among the studies included [30]. Our results also showed a substantial heterogeneity among the included trials, which was best resolved after excluding the study by Ucuncu et al. [13]. There are many reasons for heterogeneity such as different sources of shoulder pain and different durations of follow-up, inadequate patient blinding that may be vulnerable to bias, and possible placebo effects. Also, most of the studies included patients with chronic diseases, which might have impacted clinical outcomes substantially. In a study by Ucuncu et al., the design was not blinded, and placebo effect expectation was higher in the image-guided group. The study also reported that patients included were not restricted to use anti-inflammatory drugs, thus impacting clinical outcomes [13].

We evaluated changes in shoulder abduction at the sixth week, and our results indicated that image-guided steroid injection was associated with more extensive shoulder abduction than blind injection. Blooms et al. reported a difference between groups, favoring the image-guided group at one to two weeks after injection [29]. We evaluated adverse events in both groups regarding the safety profile and found no significant difference between the compared groups.

Strengths and limitations

Our study included large randomized controlled trials with moderate to high quality and large sample size, helping to understand better and evaluate the evidence clearly. We strictly adhered to the PRISMA checklist and carefully performed a precise search in the electronic database. Moreover, the mean changes between baseline and six weeks of follow-up were calculated, which offered a more accurate detection of changes at baseline. The limitations in our study included: 1) a substantial heterogeneity was observed in our analysis (I^2^ > 50%, P < 0.1), and we conducted a leave-one-out meta-analysis to assess the contribution of each study to the overall model. No study affected the overall estimate in the leave-one-out-study analysis, and there was no marked difference in results, suggesting that a single study did not drive the results of the current study. 2) There was marked variation in the functional scales used between studies (Constant-Murley Score, Oxford score, and other scores); therefore, we used SMD for all functional scores. 3) Due to the inadequate data in the literature, the correlation between injection accuracy and clinical improvement was not studied.

## Conclusions

Our meta-analysis investigated the efficacy and safety of image-guided injections compared to blind steroid injections. In terms of efficacy, the image-guided approach was associated with significant improvement in pain and shoulder functionality. However, we found no significant difference between the two approaches in terms of safety. Future studies recommend investigating and comparing the risk of adverse events associated with these approaches and correlate needle injection accuracy and the efficacy and safety of the outcomes. Also, further studies to assess the impact of operators (radiologists or non-radiologists) on the outcomes and cost-effectiveness of the two approaches are desired.
